# Optimization of the driver’s seat belt and injury biomechanical analysis in real-world minivan small offset impact accident scenarios

**DOI:** 10.3389/fbioe.2022.965206

**Published:** 2022-10-20

**Authors:** Xiuju Yang, Jingjing Shi, Qianying Fu, Shanshan Pu, Chunxiao Lian, Kui Li, Zhiyong Yin, Shengxiong Liu, Guixue Wang

**Affiliations:** ^1^ Key Laboratory for Biorheological Science and Technology of Ministry of Education, State and Local Joint Engineering Laboratory for Vascular Implants, College of Bioengineering, Chongqing University, Chongqing, China; ^2^ Department of Biomedical Engineering, College of Pharmacy and Bioengineering, Chongqing University of Technology, Chongqing, China; ^3^ Chongqing Key Laboratory of Vehicle Crash/Bio-Impact and Traffic Safety, Department 4, Institute of Surgery Research, Daping Hospital, Army Medical University, Chongqing, China; ^4^ China Automotive Engineering Research Institute Co., Ltd., Chongqing, China

**Keywords:** small offset collision, accident reconstruction, driver injury, pre-tensioned force-limiting seat belt, multi-objective genetic algorithm, biomechanics analysis, genetic algorithm

## Abstract

To minimize injuries and protect the safety of the driver in minivan small offset collisions, an optimized pre-tensioned force-limiting seat belt was proposed herein. An accident with detailed information, such as medical reports, vehicle inspection reports, and accident scene photographs, was reconstructed using HyperMesh software. The effectiveness of both the accident model and the pre-tensioned force-limiting seat belt was evaluated. To obtain the optimal seat belt parameters for driver protection, first, force-limiting A, pre-tensioned force B, and pre-tensioned time C factors were selected in designing an orthogonal test with different factor levels. The influence laws of each factor on the injury biomechanical characteristics of the driver were analyzed *via* the direct analysis method. Moreover, each kind of critical injury value of the human body was synthesized, and the radial basis function surrogate model was constructed. The three seat belt parameters were optimized using the NSGA-II multi-objective genetic algorithm. The results showed that the optimal balance variable parameter of the seat belt was 4751.618 N–2451.839 N–17.554 ms (A–B–C). Finally, the optimal scheme was verified in a system simulating a minivan small offset collision. The results showed that after optimization, the skull von Mises stress was reduced by 36.9%, and the stress of the cervical vertebra cortical bone and cancellous bone decreased by 29.1% and 30.8%, respectively. In addition, the strains of the ribs and lungs decreased by 31.2% and 30.7%, respectively.

## 1 Introduction

Minivans have a large market share in China because of their low price, large internal space, and compact engine and cab layout. The energy absorption space of the front-end of minivans is small, their body materials are made of plain carbon steel, and their safety configuration is low. Hence, the invasion of the occupants’ cabin during small offset collisions is severe, seriously threatening the safety of drivers (Li et al., 2015; Prochowski et al., 2016; Shi et al., 2016).

The safety of drivers in a minivan collision is an important issue. The structural crashworthiness and the design of the restraint systems of minibuses are the focus of current research (Hassan and Meguid, 2018; Hu et al., 2018). By contrast, the structural crashworthiness of minivans is mainly evaluated *via* numerical simulations. Moreover, previous studies concentrated on optimizing the energy absorption mechanism, characteristics, and crashworthiness of the thin-walled structures of minivans. Anghileri et al. (2005) identified the damage parameters of a model of “bi-phase” materials *via* PAM-CRASH. They determined these parameters from the dynamic axial crushing of thin-walled cylinder tubes with different fiber orientations by using an inverse method combined with the multi-objective optimization method to identify damage and failure mechanisms in composite materials. Cho et al. (2006) evaluated the crashworthiness of rectangular and circular dent-type crush initiators to analyze the influence of the ratio of wall thickness and size on crashworthiness. They found that the rectangular dent-type crush initiator absorbs more crash energy than the circular dent-type crush initiator. The crashworthiness of automobiles can be improved by using energy-absorbing structures (Park, 2011; Ovesy and Masjedi, 2014).

Given that collision forces throw the occupants out of the vehicle, researchers developed various restraint systems (Zhai et al., 2015; Tian et al., 2016; Liu et al., 2017a; Albanese et al., 2020). Previous studies primarily focused on optimizing the performance of safety belts, airbags, crash dummies, and seats to effectively avoid or reduce the secondary collision of compartment structures and occupants in minivans. Zhai et al. (2017) clarified the influence of the variable optimization of airbags, seat belts, and pretension times on the head and chest injury of occupants by using a genetic algorithm. They found that head and chest injuries can be greatly reduced by designing reasonable energy-absorbing structures and matching the restraint systems of vehicles. Liu et al. (2016) examined the influence of seat belt hang-point positions, elongations, and initial strains on occupant impact injury. They established a simulation model of seat belt restraint systems by using MADYMO software and constructed an approximate model by using the radial function. Then, they optimized the seat belt parameters. They reported that the optimized seat belts can effectively reduce the injuries of the occupants. Gu et al. (2020) simulated and optimized the anchorage of seat belts. They stated that the safety performance of vehicles can be increased, and the contact surface between the anchorage stiffener plate and the inner plate of the B-pillar can be enhanced by improving the structure of the anchorage stiffener plate. NSGA-II is an improved version of the non-dominated sorting genetic algorithm (NSGA), and it has high operation efficiency, good distribution of the solution set, good convergence, and robustness (Deb et al., 2002; Wang et al., 2020). The researchers used the NSGA-II genetic algorithm to optimize the key parameters of the occupant pre-tensioned force-limiting seat belt restraint system (Ge et al., 2017). The results show that this method can quickly and effectively obtain the optimal matching parameters of the pre-tensioned force-limiting seat belt restraint system and ensure the safety of vehicle occupants.

Simulation research on minivans is relatively perfect. However, because of the large number of calculation parameters involved, the calculation time is long and the error is large. Gao et al. (2019) compared and analyzed four approximate models of minivans, namely, response surface, radial basis neural network, Kriging polynomial, and orthogonal polynomial. They stated that replacing the real model with an approximate model can substantially reduce the calculation time and ensure the feasibility of optimization. In this study, small offset collisions were simulated, and the effect of different seat belt variables on the Total Human Model for Safety (THUMS), that is, the head, neck, thorax, and legs were examined. An accident was reconstructed using detailed crash information. An accident model was then tested to determine its effectiveness. We analyzed the effects of seat belts, including those that are commonly used and those that are pre-tensioned force-limiting, on the head, neck, thorax, and legs of drivers. On this basis, we used the NSGA-II genetic algorithm to optimize the key parameters of the pre-tensioned force-limiting seat belt. The results show that the optimized pre-tensioned force-limiting seat belt plays a good protective effect on the driver. Finally, to test the effectiveness of the optimized scheme, the optimized seat belt parameters were substituted into the simulation model.

## 2 Materials and methods

### 2.1 Accident data

Our work is supported by an in-depth accident investigation carried out by a research team at the Surgical Institute of Army Military University in Chongqing, China, who gathered data on more than 2,700 traffic incidents from 2013 to 2018 and created a database (Duan et al., 2020). To ascertain how each collision happened, pertinent information is gathered from the traffic police division. Typical small offset collision crashes relating a minivan were selected from our database for this study. This accident has detailed photos of the accident scene, police traffic accident scene map, injury report, vehicle trace deformation, and other information. The details of the accident collision process is as follows: the location of the accident is a two-way four lane, the weather is light rain, and the ground is the wet asphalt pavement; a small offset collision of 25% occurs between the left front of a minivan and the left front of a sedan; when the collision occurs, the collision velocity of the minivan is 54 km/h (Liu et al., 2017b; Wang et al., 2022a), and the driver uses the seat belt, but without the airbag. The abridged injury scale (AIS), which was amended in 2005, was used to categorize the injury information of the drivers involved in the accidents. The AIS values of 1, 2, 3, 4, 5, and 6 correspond to minor, moderate, serious, severe, critical, and untreated injuries, respectively, according to the criteria. The details of the collision, vehicle, and driver of the accident are shown in Table 1.

**TABLE 1:** Details about the collision, vehicle, and driver.


Collision information	Weather	Light rain	
	Ground	Wet asphalt	
	Collision type	25% offset collision	
	Impact velocity (km/h)	54	
Driver information	Age	38	
	Gender	Male	
	Stature (cm)	160	
Vehicle information	Vehicle type	Minivan	
	Curb weight (kg)	1,015	
	Wheelbase (mm)	2,500	
	Length × width × height (mm)	3,860 × 1,500 × 1,900	
Driver injury	Injury part	AIS	Injury information
	Head	3	Mid-frontal scattered with contusions and lacerations. Left eyebrow with arcuate wound. Depth of the wound cavity reached the muscular layer. Concave fracture of the left eyebrow arch and skull fracture
	Neck	2+	Fracture of the thyroid cartilage in the neck
	Thorax	4	Closed fracture of the sternum, rib fracture, and lung contusion
	Lower limb	3	Large open laceration on the left medial thigh. Amputation wounds were seen in both lower limbs
Extent of vehicle damage	The front windshield cracked. The hood was dented and deformed. Front bumper skin with peeled and concave deformation. The left front wheel and suspension were deformed. The left A-pillar was deformed. The left front door and the left center door were deformed. The window and sealing rubber of the left front door and the left middle door detached. Left and right front headlights detached	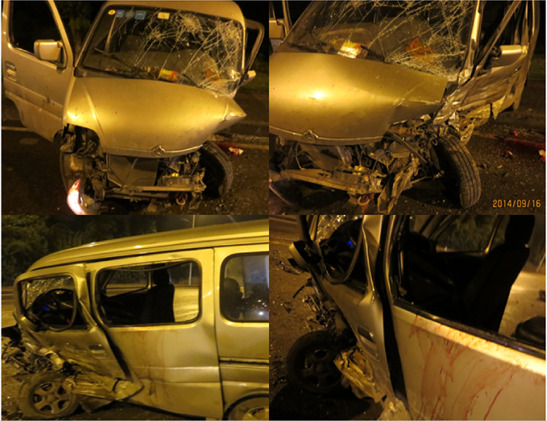	

### 2.2 Accident reconstruction

**FIGURE 1:**
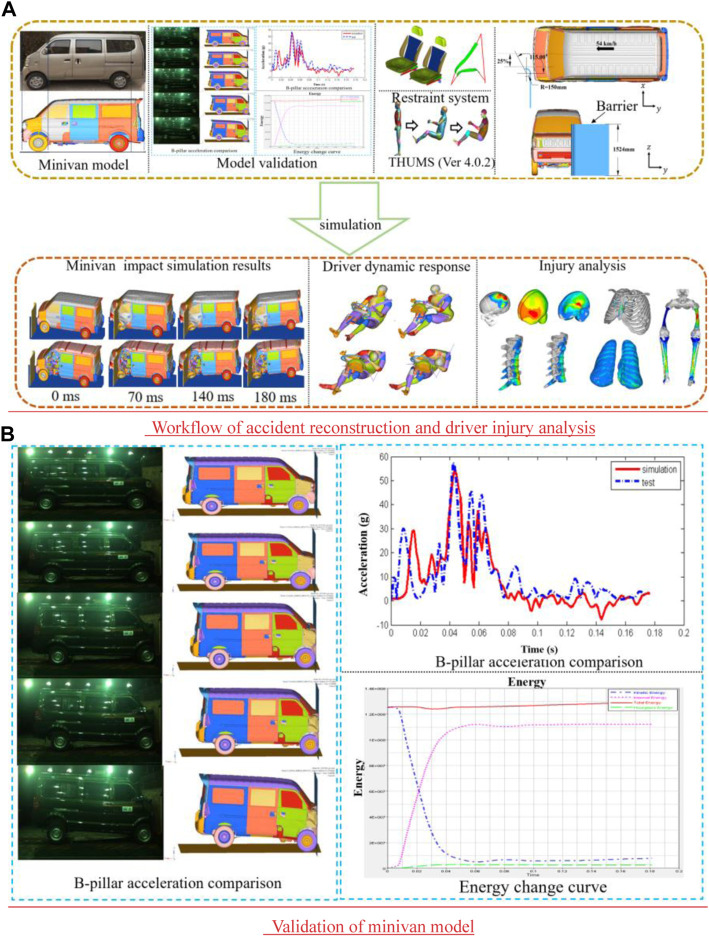
Accident reconstruction. **(A)** Workflow of accident reconstruction and driver injury analysis and **(B)** validation of the minivan model.

Figure 1A shows the workflow involved in reconstructing the accident. The THUMS model (version 4.0.2) was used to adjust the dummy position with an initial force or an initial velocity (Magee and Thornton, 1978). The vehicle finite element model was constructed based on the prototype of the minivan in this case. Vehicle models included body, windshield, seating system, steering system, dashboard, and pedals. The model had 727,826 units, and its materials and characteristics satisfied the collision standards’ fundamental criteria. Figure 1B shows that after the finite-element model of the minivan is constructed, the validity and accuracy of the model are verified. Lei and Yin (2016) conducted 100% rigid wall frontal crash experiments on real vehicles and finite-element vehicle models. The results of the real-vehicle test and simulation show that the dynamic response process of the minivan in simulation is basically consistent with the results of a real-vehicle collision. The acceleration value and change trend of the B-pillar are basically consistent, and the total energy is basically stable, which conforms to the requirements of the energy conservation law. Therefore, the finite-element model of the minivan can effectively simulate and analyze the small offset collision. In accordance with China Insurance Automotive Safety Index (C-IASI) regulations, a rigid barrier with a simplified arc structure was adopted in this work. The height of the barrier was 1,524 mm, the radian of the arc surface was 115°, and the radius of the arc surface was 150 mm. Based on the performance of the seat belt in the crash vehicle, the ordinary three-point seat belt model was established.

A boundary condition included the relative position of the minivan to the barrier as well as the ground impact. Using the crash information, the contact location seen between the vehicle and the barrier was set, and the barrier was completely constrained. The barrier is situated on the left side of the minivan body, and there is a 25% overlap rate with the vehicle body. The vehicle acted as the slave surface, the barrier acted as the main surface, the dynamic friction coefficient was 0.1, and the static friction coefficient was 0.2. The type of contact between the THUMS model and the vehicle interior was defined as automatic face-to-face contact (Wang et al., 2022b). An asphalt pavement, which is considered a rigid body, was used in the test (Hu et al., 2011). The wet asphalt pavement had a friction coefficient of 0.3. The vehicle impact velocity was set at 54 km/h.

### 2.3 Establishment of the seat belt finite-element model

#### 2.3.1 Establishment of the finite-element model of the ordinary seat belt

A seat belt, which protects the vehicle occupants in the event of a crash, is an effective, compulsory safety device in vehicle restraint systems. The most commonly used type is a three-point seat belt consisting of a seat belt fixed at one end, a D-ring, and a retractor. In this study, an ordinary three-point seat belt, which mainly includes a seat belt webbing, a rewinding device, a D-ring, and a seat belt anchorage, was modeled using Primer software. The 1D seat belt unit and the 2D shell unit comprised the seat belt webbing.

#### 2.3.2 Establishment of a finite-element model of a pre-tensioned force-limiting seat belt

**FIGURE 2:**
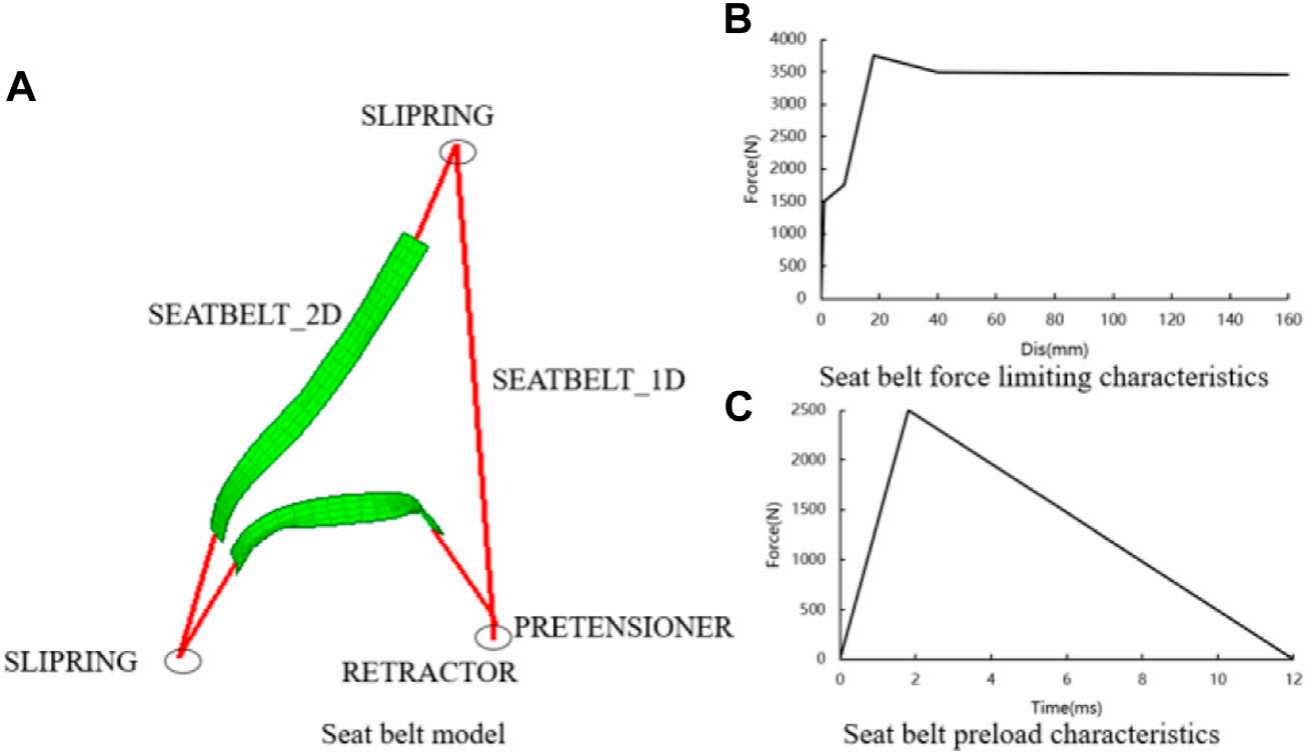
Pre-tensioned force-limiting seat belt. **(A)** composition of pre-tensioned force-limiting seatbelt, **(B)** force limiting characteristic curve of seatbelt, and **(C)** pre-tightening curve of seatbelt.

By the definition of the belt webbing using the card *ELEMENT_SEATBELT_RETRACTOR in Primer software, the force-limiting seat belt was implemented. A pre-tensioner is a device that controls the retraction part of the seat belt using a rewinding device. The pre-tensioned function was realized through the card *ELEMENT_SEATBLET_PRETENSIONER definition (Figure 2).

The police investigation found that the minivan was not equipped with airbags. Seat belts are the most important restraint systems. To reduce the risk of injury to the driver, the model of the pre-tensioned force-limiting seat belt was optimized for three variables. Limiting forces A, pre-tensioned forces B, and pre-tensioned times C were the design variables. Those variables were designed in the range of 4500 N ≤ A ≤ 5500 N, 1500 N ≤ B ≤ 2500 N, and 16 ms ≤ C ≤ 24 ms, respectively.

### 2.4 Injury criteria

The degree of head injury is evaluated using the head injury criterion (HIC). As a measure of head injury, the HIC is widely accepted and used. The theoretical expression is as follows (Marjoux et al., 2008):

$$HIC=\max_{T_{0}\leq t_{1}\leq t_{2}\leq T_{E}}\left\{\left(t_{2}-t_{1}\right){\left[\frac{1}{t_{2}-t_{1}}\overset{t_{2}}{\underset{t_{1}}{\int }}a\left(t\right)dt\right]}^{2.5}\right\}.$$ (1)

In this expression, $T_{0}$ represents the beginning of the simulation and $T_{E}$ represents the end of the simulation. The starting time and the ending time when the HIC reaches its maximum value are indicated by $t_{1}$ and $t_{2}$ , respectively. In the integral time throughout the collision, a(t) represents the combined acceleration of the head in the X, Y, and Z directions. A head injury value is the maximum calculated in the integral time throughout the collision. HIC_15_, i.e., *t*_*2*_−*t*_*1*_ = 15 ms, was used for small offset collisions in the 2018 China-New Car Assessment Programme (C-NCAP).

Based on the THUMS with high biological fidelity, three biomechanical parameters were used to analyze the head injury of drivers. They were skull von Mises stress, intracranial pressure, and intracranial von Mises stress. In addition, the tolerance limits were 10 MPa, 235 KPa, and 15–20 kPa, respectively (Chan and Liu, 1974; Willinger et al., 2000).

The biomechanical neck injury ( $N_{ij}$ ) predictor is a useful index for determining the severity of occupant injuries. $N_{ij}$ is calculated as follows:

$$N_{ij}=\left|\frac{F_{z}}{F_{zc}}\right|+\left|\frac{M_{ocy}}{M_{yc}}\right|,$$ (2)

where $F_{z}$ denotes the axial force and $M_{ocy}$ denotes the bending moment. The critical intercept values $F_{zc}$ and $M_{yc}$ correspond to the axial force and the neck bending moment, respectively. In collision accidents, the FMVSS208 standard specifies that the $N_{ij}$ tolerance limit value is 1 (Wen et al., 2011).

Cervical cortical bone stress and cancellous bone stress were used to determine the extent of neck tissue injury. Studies have shown that cervical vertebra fractures occur when cortical bone stress and cancellous bone stress exceed 236 and 59 MPa, respectively (Chi and Geng, 2019).

The contiguous 3 ms injury criteria and maximal thorax compression are important indices for occupant chest injury in frontal collisions. According to FMVSS208, the maximal resultant linear acceleration of a thorax during a continuous span of 3 ms cannot surpass 60 g, i.e., $C_{3ms}$ ≤ 60 g (Marjoux et al., 2008). When the chest compression reaches 55 mm, there is a 50% probability of rib fracture (Mertz et al., 1997). Rib and lung strains are also used to evaluate the driver’s chest injury. Studies have shown a risk of fracture when the rib strain exceeds 3%, and the threshold of lung strain is 30% (Schaefer et al., 1958; Kallieris et al., 1997).

In this study, leg injury was evaluated by the axial force of the thigh $F_{femur}$ , femur, and tibia strain. The study showed that the axial force of the thigh should not exceed 10 KN (Viano and Arepally, 1990), and the strain of the femur and tibia would be more than 3% (Kuppa et al., 2001).

In small offset collisions, drivers suffer from different degrees of injury to various body parts. The degree of injury to the human body was evaluated. To evaluate the injuries to the head, thorax, and legs comprehensively, the weighed injury criteria (WIC) were used. The WIC are calculated as follows (Viano and Arepally, 1990):

$$WIC=0.6\left(\frac{{HIC}_{15}}{700}\right)+\frac{0.35\left(\frac{C_{3ms}}{60}+\frac{C_{comp}}{63}\right)}{2}+0.05\left(\frac{F_{L}+F_{R}}{20}\right),$$ (3)

where $C_{comp}$ denotes the compressive amount of the thorax and $F_{L}$ is the maximum axial force for the left thigh bone, whereas $F_{R}$ is the maximum axial force for the right thigh bone.

## 3 Results and discussion

### 3.1 Validation of the accident model

**FIGURE 3:**
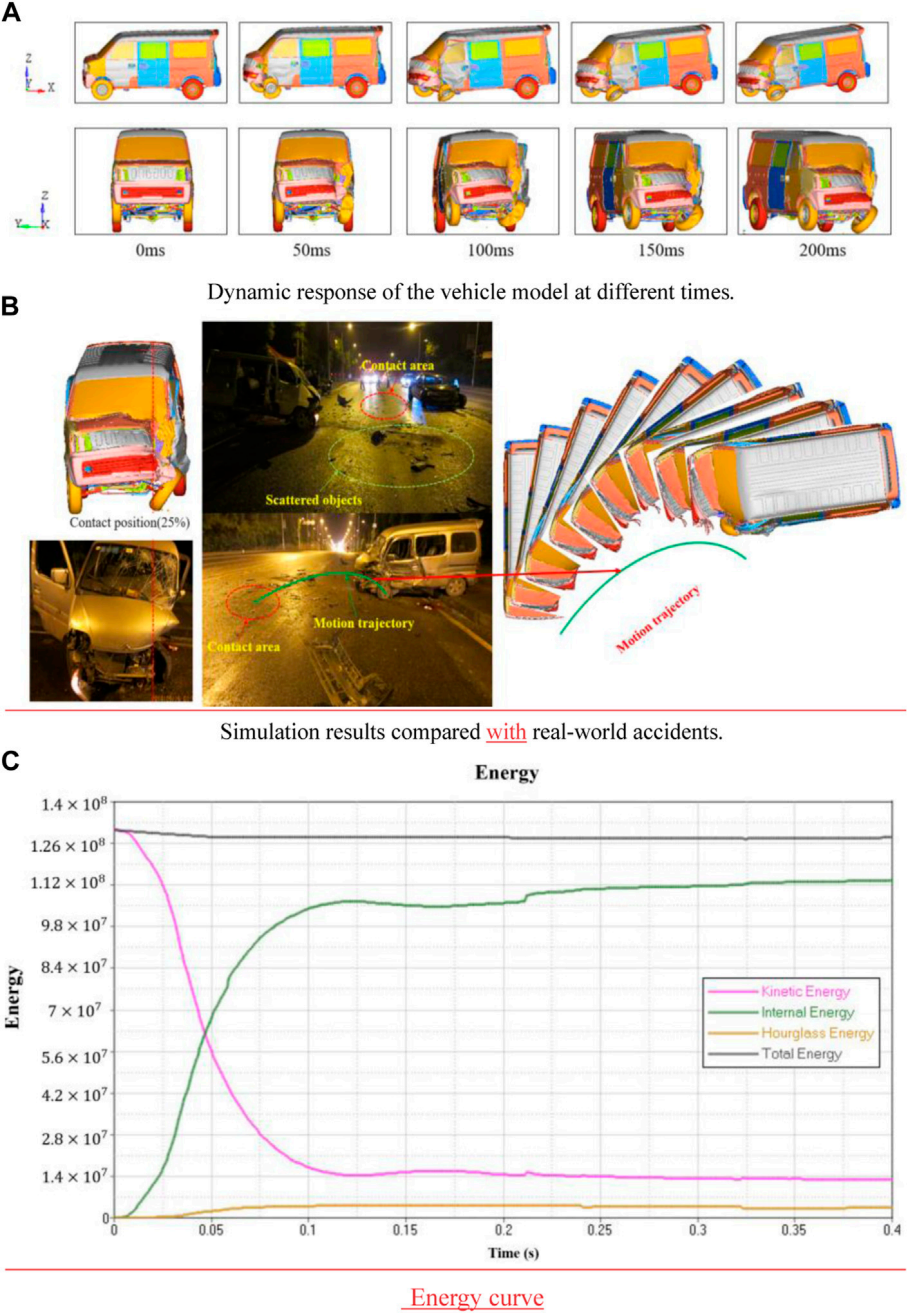
Dynamic response of the vehicle model and comparison between the simulation results and real-world accidents. **(A)** Dynamic response of the vehicle model at different times. **(B)** Simulation results compared with real-world accidents. **(C)** Energy curve.

The simulation of the changes that occurred in the minivan during the small offset collision is outlined in Figure 3A. The deformation of the vehicle was observed at different times. During the small offset collision, the front windshield broke, and the hood, the front bumper, the left front wheel, the suspension, and the left A-pillar were deformed. The simulation results were consistent with the actual deformation of the vehicle during the accident. The vehicle model and the barrier contact positions were consistent with accident vehicle collision positions as shown in Figure 3B. The impacted area of the vehicle was inferred from the main concentrated area of the scattered objects at the accident site and the final stopping position of the vehicle. The motion track of the vehicle at the time of the accident was obtained. In accordance with the observations of real-world accidents, the reconstructed collision track had a good correlation with the accident collision track. The energy change curve of the minivan during the small offset collision can be used as an important index to evaluate the reliability of the simulation results. Figure 3C illustrates that the simulation model is in an energy conservation state during the whole collision process, with a smooth curve and small fluctuation. The conversion between kinetic energy and internal energy and the ratio of hourglass energy to total energy are all within the acceptable 5% range. Therefore, the aforementioned results verify the effectiveness of the collision model.

**FIGURE 4:**
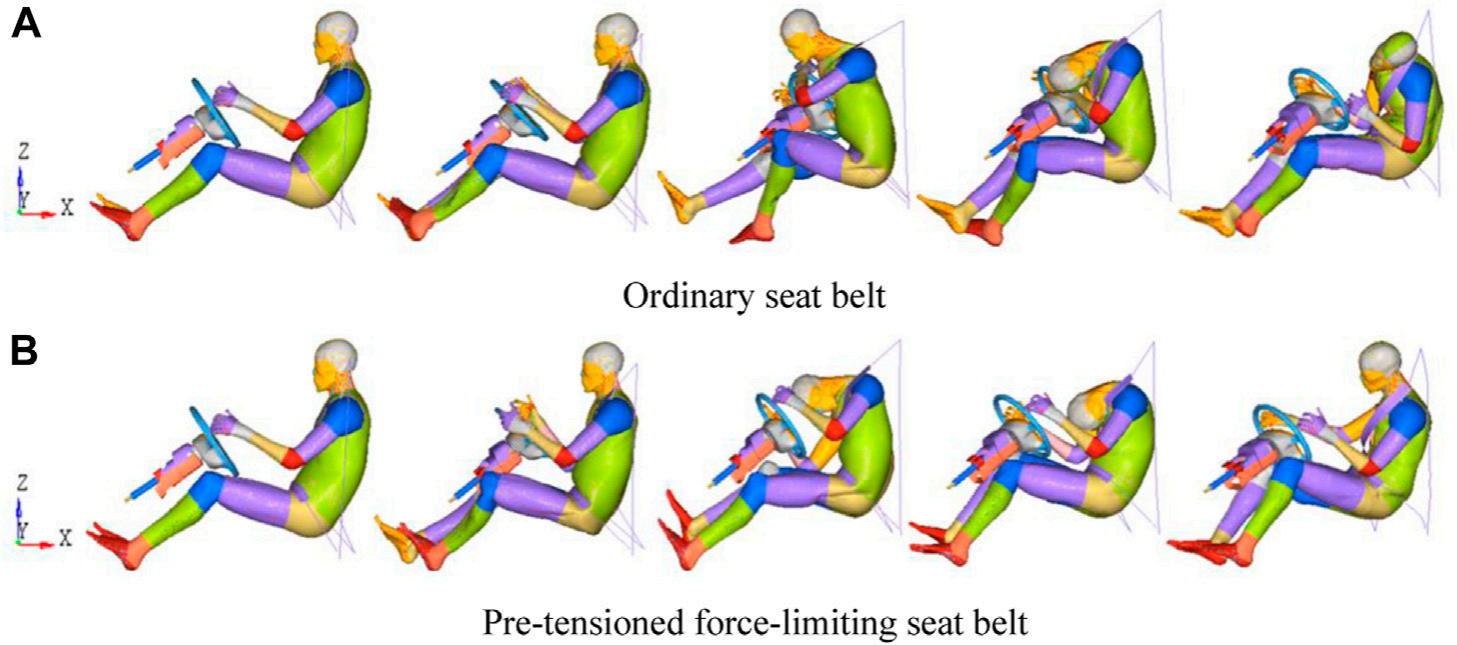
Dynamic reaction of a dummy wearing an ordinary seat belt and a dummy wearing a pre-tensioned force-limiting seat belt. **(A)** Ordinary seat belt. **(B)** Pre-tensioned force-limiting seat belt.

The dynamic reaction of a driver wearing an ordinary seat belt and another driver wearing a pre-tensioned force-limiting seat belt was compared to identify the differences between the pre-tensioned force-limiting seat belt and the ordinary seat belt (Figure 4). During the entire period of the collision, the dummy moved forward and toward the left because of longitudinal deceleration and the acceleration force along the *Z* direction. The dummy’s knees first came into contact with the vehicle’s interior after the collision, as shown in Figure 4A. The dummy then continuously moved forward and toward the left because of the effects of inertia. The chest hit the steering wheel. Finally, the dummy stopped moving forward because of the tension of the seat belt. The legs of the dummy were first hit by the vehicle’s interior, as shown in Figure 4B. The pre-tensioned force-limiting seat belt prevented the direct violent collision between the chest and the steering wheel. Moreover, the seat belt shortened the distance moved by the head as it leaned forward, thereby protecting the head.

According to the dynamic response of the THUMS dummies, 14 injury indexes were used to evaluate the risk of injury to the head, thorax, and legs. The amount of protection provided by the ordinary seat belt and the pre-tensioned force-limiting seat belt against the injuries to the dummies’ head, neck, chest, and legs was compared (Table 2).

**TABLE 2:** Injury values for dummies’ heads, necks, chests, and legs.

	Injury criteria	Injury threshold	Ordinary seat belt	Pre-tensioned force-limiting seat belt	Reduction (%)
Head	Skull von Mises stress (MPa)	10	31.8	23.6	25.8
	Intracranial pressure (kPa)	235	277.8	198.7	28.5
	Intracranial von Mises stress (kPa)	15–20	19.1	14.6	23.6
	HIC	700	834.6	521.3	37.5
Neck	$N_{ij}$	1	1.24	0.87	29.8
	Cortical bone stress (MPa)	236	159.8	123.4	22.8
	Cancellous bone stress (MPa)	59	16.3	11.7	28.2
Chest	$C_{3ms}$ (g)	60	103.4	92.4	10.6
	$C_{comp}$ (mm)	50	45.3	37.8	16.6
	Rib strain	3%	43.4%	32.4%	25.3
	Lung strain	30%	29.2%	22.5%	22.9
Leg	$F_{L}$ (KN)	10	15.5	13.2	14.8
	$F_{R}$ (KN)	10	12.1	10.5	13.2
	Femur strain	3%	6.1%	4.9%	19.7
	Tibia strain	3%	9.7%	8.3%	14.4
WIC		1	1.212	0.881	27.3

The simulation results indicated that the HIC, von Mises stress, intracranial pressure, and intracranial von Mises stress with ordinary seat belts were larger than the injury thresholds, a condition that may lead to skull fracture and serious injury. This mostly agrees with the driver’s head injury, which is listed as AIS 3 in Table 1. These injuries may have been caused as the driver’s head hit the windshield, the steering wheel, and the instrument panel, consistent with the report of Pappachan and Alexander (2012). Injury values of $N_{ij}$ , cortical bone, cancellous bone stress, chest ( $C_{3ms}$ ), ribs, lungs, heart, and liver were larger than the injury thresholds, a condition that may lead to serious neck and chest injuries. This mostly agrees with the driver’s AIS 2+ neck injury and AIS 4 chest injury noted in Table 1 (Hao et al., 2018; Lu et al., 2016). These injuries might have been caused as the chest was restrained by the seat belt and hit the steering wheel. The maximum axial force of the driver’s left and right legs was 13.5 KN and 12.1 KN, respectively. That means the driver’s left thigh was more seriously injured. A strain of 6.8% on the femur and 9.7% on the tibia, above the injury threshold, shows that the driver suffered serious injury to his lower extremities. The observation was consistent with the injury report: large open laceration on the left medial thigh. Amputation wounds were seen in both lower limbs. It is due to the fact that the impact region is on the vehicle’s front-left side and is heavily contaminated, and the left lower limb is in contact with the A-pillar lower hinge, sill, and foot pedal (xiao et al., 2018).

The results of the comparison of vehicle deformations and the driver’s injuries verified the credibility of the crash simulation model, consistent with the results obtained by Shigeta et al. (2009) and Watanabe et al. (2012).

The pre-tensioned force-limiting seat belts reduced the chances of head, neck, chest, and leg injuries compared with the ordinary seat belts, as shown in Table 3. Therefore, pre-tensioned force-limiting seat belts offer better protection for the driver.

**TABLE 3:** Simulation results of the driver’s head injury value.

Index	HIC			Skull von Mises stress (MPa)			Intracranial pressure (kPa)			Intracranial von Mises stress (kPa)		
	A	B	C	A	B	C	A	B	C	A	B	C
K1	3,092.6	3,178.6	2,978.8	143.9	156.1	145.4	1,191.9	1,227.1	1,135.5	95.5	89.6	81.9
K2	2,720.8	3,384.6	3,161.3	127.9	164.9	153.1	1,046.2	1,301.2	1,121.7	81.8	96.6	89.3
K3	3,293.5	3,573.8	3,586.4	168.9	172.1	171.6	1,245.9	1,275.6	1,291.7	92.2	101.1	101.0
K4	3,780.9	3,376.9	3,380.0	186.9	162.8	162.3	1,441.4	1,192.6	1,200.0	103.1	95.0	95.9
K5	3,727.2	3,101.1	3,508.5	178.2	149.9	173.4	1,176.4	1,105.3	1,352.9	96.0	86.3	100.5
k1	618.52	635.72	595.76	28.78	31.22	29.08	238.38	245.42	227.10	19.10	17.92	16.38
k2	544.16	676.92	632.26	25.58	32.98	30.62	209.24	260.24	224.34	16.36	19.32	17.86
k3	658.70	714.76	717.28	33.78	34.42	34.32	249.18	255.12	258.34	18.44	20.22	20.20
k4	756.18	675.38	676.00	37.38	32.56	32.46	288.28	238.52	240.00	20.62	19.00	19.18
k5	745.44	620.22	701.70	35.64	29.98	34.68	235.28	221.06	270.58	19.20	17.26	20.10
R	**212.02**	94.54	121.52	**11.8**	4.44	5.60	**79.04**	39.18	46.24	**4.26**	2.96	3.82

The meaning of the bold value we provide is to highlight the highest value of R among the three variables A, B and C in the text.

### 3.2 Effects of different parameters of pre-tensioned force-limiting seat belts on driver injury

**FIGURE 5:**
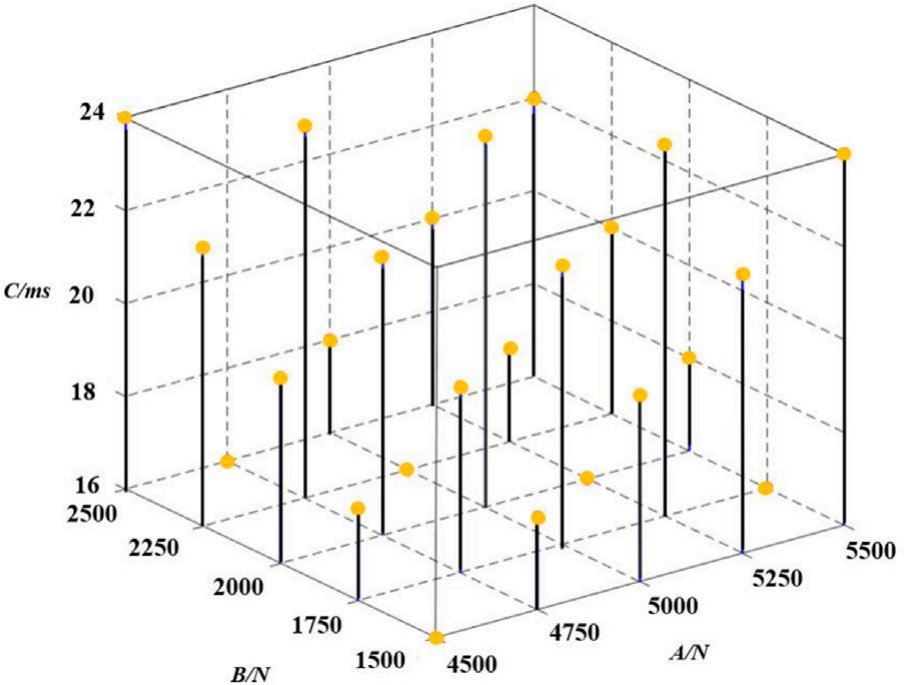
Sample distribution resulting from an orthogonal experimental design.

Further improved safety protection was provided to the driver by the pre-tensioned force-limiting seat belt. The three variables with a great influence on the restraint performance of the pre-tensioned force-limiting seat belt, namely, seat belt force-limiting A, pre-tensioned force B, and pre-tensioned time C, were optimized. With the three factors at 5 levels and 25 samples, an orthogonal experimental design was applied. Figure 5 shows how the sample points are distributed spatially.

The injuries of the drivers in different seat belt variables were analyzed *via* the direct analysis method. Various design variables were examined for their influences on the dummy injuries. Tables 3–Tables 6 show that the sum of the test results across factors and levels is represented by Ki (i = 1, 2, 3, 4, and 5). The average of the test results under each factor and level is ki (i = 1, 2, 3, 4, and 5). An indication of the range of test results within the same factor and at various levels is R.

**TABLE 4:** Simulation results of the driver’s neck injury value.

Index	$N_{ij}$			Cortical bone stress (MPa)			Cancellous bone stress (MPa)		
	A	B	C	A	B	C	A	B	C
K1	4.48	5.31	4.01	642.7	759.2	572.8	60.1	70.5	52.6
K2	4.02	4.65	3.93	580.5	665.8	564.4	54.7	61.9	52.7
K3	4.11	4.18	4.55	589.4	601.3	655.7	55.2	55.9	60.5
K4	4.52	4.28	4.70	643.9	616.5	673.9	59.6	57.4	62.7
K5	4.90	3.61	4.84	703.9	517.6	693.6	63.7	47.6	64.8
k1	0.896	1.062	0.802	128.54	151.84	114.56	12.02	14.10	10.52
k2	0.804	0.930	0.786	116.10	133.16	112.88	10.94	12.38	10.54
k3	0.822	0.836	0.910	117.88	120.26	131.14	11.04	11.18	12.10
k4	0.904	0.856	0.940	128.78	123.30	134.78	11.92	11.48	12.54
k5	0.980	0.722	0.968	140.78	103.52	138.72	12.74	9.52	12.96
R	0.176	**0.34**	0.182	24.68	**48.32**	25.84	1.80	**4.58**	2.44

The meaning of the bold value we provide is to highlight the highest value of R among the three variables A, B and C in the text.

**TABLE 5:** Simulation results of the driver’s chest injury value.

Index	$C_{comp}$ (mm)			$C_{3ms}$			Rib strain			Lung strain		
	A	B	C	A	B	C	A	B	C	A	B	C
K1	187.7	165.8	151.7	466.3	422.7	387.1	164.1%	145.8%	133.2%	113.4%	102.8%	94.5%
K2	171.2	172.8	140.7	428.1	439.5	359.6	147.9%	150.9%	124.1%	103.5%	106.9%	87.1%
K3	163.9	176.2	179.6	419.2	449.3	453.5	143.5%	155.2%	155.7%	101.7%	109.1%	110.8%
K4	165.3	158.8	185.6	430.8	402.1	468.0	147.6%	138.4%	162.1%	105.1%	98.0%	113.6%
K5	160.8	175.3	191.3	415.1	445.9	491.3	140.6%	153.4%	168.6%	101.6%	108.5%	119.3%
k1	37.54	33.16	30.34	93.26	84.54	77.42	32.82%	29.16%	26.64%	22.68%	20.56%	18.90%
k2	34.24	34.56	28.14	85.62	87.90	71.92	29.58%	30.18%	24.82%	20.70%	21.38%	17.42%
k3	32.78	35.24	35.92	83.84	89.86	90.70	28.70%	31.04%	31.14%	20.34%	21.82%	22.16%
k4	33.06	31.76	37.12	86.16	80.42	93.60	29.52%	27.68%	32.42%	21.02%	19.60%	22.72%
k5	32.16	35.06	38.26	83.02	89.18	98.26	28.12%	30.68%	33.72%	20.32%	21.70%	23.86%
R	5.38	3.48	**10.12**	10.24	9.44	**26.34**	4.70%	3.36%	**8.90%**	2.48%	2.22%	**6.44%**

The meaning of the bold value we provide is to highlight the highest value of R among the three variables A, B and C in the text.

**TABLE 6:** Simulation results of the driver’s leg injury value.

Index	$F_{femur}$ (KN)			Femur strain			Tibia strain		
	A	B	C	A	B	C	A	B	C
K1	64.7	65.9	52.6	24.1%	24.9%	19.8%	41.0%	42.9%	34.5%
K2	44.3	59.2	55.5	16.8%	22.3%	21.0%	29.1%	38.7%	36.2%
K3	52.0	55.8	61.6	19.9%	21.0%	23.4%	34.3%	36.3%	40.1%
K4	64.1	49.8	55.9	24.3%	18.9%	21.1%	42.3%	32.7%	36.7%
K5	53.0	47.4	52.5	20.2%	18.2%	20.0%	35.1%	31.2%	34.3%
k1	12.94	13.18	10.52	4.82%	4.98%	3.96%	8.20%	8.58%	6.90%
k2	8.86	11.84	11.10	3.36%	4.46%	4.20%	5.82%	7.74%	7.24%
k3	10.40	11.16	12.32	3.98%	4.20%	4.68%	6.86%	7.26%	8.02%
k4	12.82	9.96	11.18	4.86%	3.78%	4.22%	8.46%	6.54%	7.34%
k5	10.60	9.48	10.50	4.04%	3.64%	4.00%	7.02%	6.24%	6.86%
R	**4.08**	3.7	1.82	**1.50%**	1.34%	0.72%	**2.64%**	2.34%	1.16%

The change in seat belt force-limiting A would have the largest effect just on the head and legs, whereas the strongest influence on the neck was caused by the change in seat belt pre-tensioning force B, according to a comparison of the range R. The change in seat belt pre-tensioned time C had the largest influence on the chest. The head and the chest were the least sensitive to changes in seat belt pre-tensioned force B, whereas the neck injury values and leg injury values were the lowest affected by seat belt force-limiting A and pre-tensioned time C, respectively.

The effect extent of each factor on the driver’s head, neck, chest, and leg injury was determined using the range analysis findings of the orthogonal experimental design to comprehend the effect of varying degrees of each element on the driver’s injury more intuitively.

**FIGURE 6:**
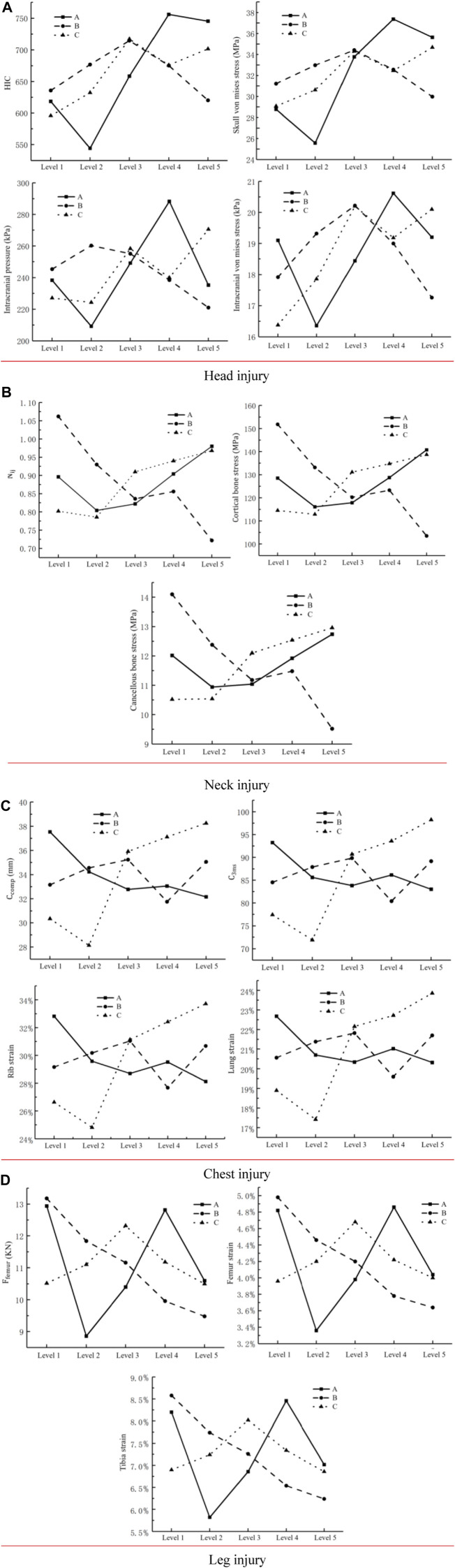
Pre-tensioned force-limiting seat belt variables’ effect laws on head, neck, thorax, and leg injuries. **(A)** Head injury. **(B)** Neck injury. **(C)** Chest injury. **(D)** Leg injury.

Figure 6 as shows the pretensioned force-limiting seat belt variables' effect laws on head, neck, thorax, and leg injuries. As shown in Figure 6A, seat belt force-limiting A increased, the injury values of the head, chest, and leg of the drivers first decreased, then increased, and again decreased, and the neck injury decreased first and then increased. When force-limiting A was larger than 4,750 N, the head, neck, and leg injuries were at their lowest degrees. The lowest value of chest injury occurred when the limiting force A was 5,500 N. As seat belt pre-tensioned force B was increased, the severity of injuries to the neck and legs improved. A head injury’s value increased first and then decreased, but a chest injury’s value decreased first, then increased, and eventually decreased. When pre-tensioned force B was 2,000 N, the driver’s chest injury value rapidly declined before abruptly increasing. The lowest injury value for the chest was 2,250 N. The lowest injury values for the head, neck, and leg occurred when pre-tensioned force B was 2,500 N. As pre-tensioned time C continuously increased, the injury values of the neck and chest decreased and then increased. However, the change trend of leg injury was the opposite. The value of the driver’s head injury increased first, then decreased, and again increased. Except for intracranial pressure, the lowest value of head injury occurred when pre-tensioned time C was 16 ms. When pre-tensioned time C was 18 ms, the neck (except for cancellous bone stress) and chest injury values were the lowest. Except for the femur strain, the lowest value of leg injury occurred when preload time C was 24 ms.

When one injury objective achieved the ideal value, the other injury objective presumably reached the worst condition, according to the reaction laws of each injury value to the variables of the pre-tensioned force-limiting seat belt. Thus, drivers could not reach the optimal injury value. Through the use of Isight optimization software, the multi-objective variables of the drivers were simultaneously optimized, and the best compromised seat belt variables were derived.

## 4 Multi-objective optimization of the pre-tensioned force-limiting seat belt

Based on comprehensive consideration, the WIC for drivers’ heads, thoraxes, and legs were chosen, with neck injury predictor $N_{ij}$ as the design goal. Additionally, seat belt force-limiting A, pre-tensioned force B, and pre-tensioned time C were employed as design variables. To construct the model, sample points were selected using the orthogonal test design method.

### 4.1 Approximate model of radial basis function and error analysis

Isight optimization software was used to construct an RBF model to explore the relationship between WIC and $N_{ij}$ as well as the force-limiting A, pre-tensioned force B, and pre-tensioned time C of the seat belt (Yin et al., 2014). The approximate definition of the response function of the design variable is

$$Y\left(X\right)=\tilde{y}\left(x\right)+\varepsilon =\left(\overset{N}{\underset{i=1}{\sum }}a_{i}g_{i}\left({\left\|x-x_{i}\right\|}^{c_{i}}+a_{N+1}\right)\right)+\varepsilon ,$$ (4)

where the approximate function of the objective is $\tilde{y}\left(x\right)$ . ε denotes the relative error (RE) between the approximate and true values. $g_{i}$ is the basis function whose number of terms is indicated by N. $x-x_{i}$ represents the Euclidean distance of the basis function, whereas the undetermined coefficient is represented by $a_{i}$ . The shape parameter is indicated by $c_{i}$ .

The response surface fitted by the RBF model was assessed using the relative error (RE), maximum error (ME), root mean square error (RMSE), and coefficient of determination ( $R^{2}$ ) to determine its plausibility. The specific formula is as follows:

$$RE=100\%\times \frac{\left|\tilde{y_{i}}-y_{i}\right|}{y_{i}},$$ (5)

$$ME=100\%\times \left(\tilde{y_{i}}-y_{i}\right),$$ (6)

$$RMSE=\sqrt{\frac{\sum_{i=1}^{M}{\left(\tilde{y_{i}}-y_{i}\right)}^{2}}{M}},$$ (7)

$$R^{2}=1-\frac{SSE}{SST}=1-\frac{\sum_{i=1}^{m}{\left(\tilde{y_{i}}-y_{i}\right)}^{2}}{\sum_{i=1}^{m}{\left(y_{i}-\bar{y}\right)}^{2}},$$ (8)

where M indicates the number of sampled data points used for testing the model’s accuracy. $\tilde{y_{i}}$ represents the predicted value of the model, whereas $y_{i}$ represents the simulation analysis value of the response. The average value of the sample in the simulation analysis is $\bar{y}$ . The $R^{2}$ determination coefficient is in the range of (0, 1). As the value approaches 1, the more accurate the model becomes. RE, ME, and RMSE maximum allowable errors are limited to 0.2, 0.3, and 0.2 in this model, respectively, and the smaller the value is, the better the plausibility of the model is. An analysis of 25 sample points was conducted. Table 7 shows that the built RBF response surface model has high accuracy and may be utilized to investigate multi-objective optimization issues further.

**TABLE 7:** Error estimation of the response surface model.

Response	RE	ME	RMSE	$R^{2}$
WIC	1.58959E-14	4.73414E-14	1.93778E-14	0.9982
Nij	1.02088E-14	2.74912E-14	1.22938E-14	0.9923

### 4.2 Multi-objective optimization model

In this work, the mathematical model of multi-objective optimization of the relationship between weighted injury criterion, neck injury predictor, and key parameters is established as follows:

$$\left\{ \begin{array}{c} \min WIC=WIC\left(A,B,C\right),\\ \min N_{ij}=N_{ij}\left(A,B,C\right),\\ s.t.\;4500N\leq A\leq 5500N,\\ 1500N\leq B\leq 2500N,\\ 16ms\leq C\leq 24ms. \end{array}\right.$$ (9)

**FIGURE 7:**
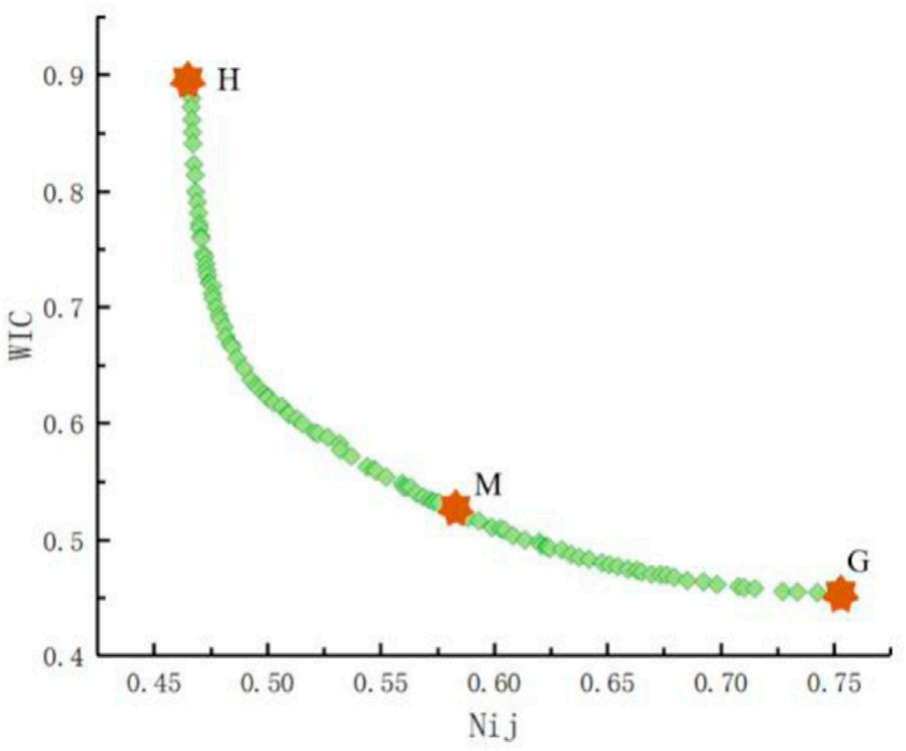
Pareto frontier of the optimal solutions of the parameters of the pre-tensioned force-limiting seat belt.

Figure 7 shows that the multi-objective genetic algorithm NSGA-II was used to obtain the multi-objective Pareto frontier within the optimal solutions of pre-tensioned force-limiting seat belt parameters (Cao et al., 2016). The optimal solutions of WIC and $N_{ij}$ may be found from the response surface model, as shown in the figure. In addition, WIC and $N_{ij}$ always demonstrated an inverse relationship, that is, a conflict existed between $N_{ij}$ and WIC, and the optimal solution could not be obtained at the same time.

In Figure 7, the *H* point represents the minimum value of $N_{ij}$ (0.455), whereas the *G* point denotes the minimum value of WIC (0.466). G and H are the two best single objective values corresponding to the different response surface models in the Pareto frontier. In this study, the method of distance minimization was used to obtain the optimal value of the weighted injury criterion and neck injury predictor. The optimal values of the neck injury predictor and the weighted injury criterion were obtained by identifying the balance between $N_{ij}$ and WIC in the optimization so that each body part was only slightly injured. The comprehensive optimal point was found when the criterion of minimizing the sum of the distances from the two Pareto response values to the optimal point to the corresponding minimum values in the solution set was the least, according to the principle of distance minimization (Yang et al., 2019). The calculation formula is as follows:

$$minZ=\left(\overset{n}{\underset{i=1}{\sum }}{\left(f_{i}^{k}-\min\left(f_{i}\right)\right)}^{2}\right),$$ (10)

where *n* = 2 denotes the number of optimization goals, i.e., the two WIC and $N_{ij}$ optimization objectives; $f_{i}^{k}$ is the response value of the *i*th optimization objective at the *k*th Pareto response point. The M value in Figure 7 is the equilibrium solution of the conflict between the two responses, based on the multi-objective optimal solution sets calculated from Eq. 10. The response model computed a neck injury ( $N_{ij}$ ) value of 0.560 at the equilibrium point M, and the WIC value was 0.545 (A = 4751.618 N, B = 2,451.839 N, and C = 17.554 ms).

### 4.3 Results

The optimal parameter values (4751.618–2,451.839–17.554) obtained from the optimization results were substituted into the crash simulation model for verification calculations. The simulation results were $N_{ij}=$ 0.589 and WIC = 0.572, and the errors were 4.92% and 4.72%, respectively. Based on the aforementioned data, all errors were determined to be within a reasonable range, which further confirmed the accuracy of the surrogate model. The optimized results were then compared with the previous simulation results, as shown in Table 8.

**TABLE 8:** Comparisons of the pre-tensioned force-limiting seat belt before and after optimization.

	Injury criteria	Before	After	Reduction (%)
Head	Skull von Mises stress (MPa)	23.6	14.9	36.9
	Intracranial pressure (kPa)	198.7	133.8	32.7
	Intracranial von Mises stress (kPa)	14.6	9.3	36.3
	HIC	521.3	328.5	37.5
Neck	$N_{ij}$	0.87	0.589	32.3
	Cortical bone stress (MPa)	123.4	87.5	29.1
	Cancellous bone stress (MPa)	11.7	8.1	30.8
Chest	$C_{3ms}$ (g)	92.4	58.2	37.0
	$C_{comp}$ (mm)	37.8	23.7	37.3
	Rib strain	32.4%	22.3%	31.2
	Lung strain	22.5%	15.6	30.7
Leg	$F_{L}$ (KN)	13.2	12.1	8.3
	$F_{R}$ (KN)	10.5	9.7	7.6
	Femur strain	4.9%	4.4%	10.2
	Tibia strain	8.3%	7.6%	8.4
WIC		0.881	0.572	35.1

The optimized pre-tensioned force-limiting seat belt protected the head, neck, thorax, and legs, as well as the entire weighted injury. Based on the injury indexes of each key part of the driver and weighted injury indexes in the optimized pre-tensioned force-limiting seat belt, the optimized pre-tensioned force-limiting seat belt was proven to be more protective and effective.

## 5 Limitations and future work

There are still certain restrictions to the present research. First of all, although the verification results of the reconstruction of the small offset collision of the minivan in this study are ideal, there will still be some errors due to the lack of video information at the time of the accident. Later accident reconstruction is expected to collect small offset collisions with video information. Second, this study only qualitatively analyzed the injury caused to the minivan driver using the pre-tensioned force-limiting seat belt. In the next step, it is necessary to study the optimization of the matching parameters between the pre-tensioned force-limiting seat belt and the airbag, so as to maximize the security value of the driver. Finally, this study only studied the protection of the minivan driver. Future research can focus on the safety performance of the occupant restraint system to reduce the risk of occupant injury.

## 6 Conclusion

The results show that in a small offset collision at 48 km/h, the pre-tensioned force-limiting seat belt with variables of 4751.618–2,451.839–17.554 had an evident protective effect on the driver’s head, neck, chest, and leg injuries, as well as the whole weighted injury. Therefore, the optimized pre-tensioned force-limiting seat belt can help drivers reach the optimal protection state in minivans when involved in small offset collisions.

(1) By reconstructing a small offset collision involving a minivan, the risk of injury to each part of the driver’s body was evaluated. Based on the results of the simulation, the model can properly portray the drivers’ dynamic characteristics as well as effectively evaluate the danger of injury to specific parts of the driver. Therefore, the crash simulation model was verified feasible.
(2) As pre-tensioned force B changed, it profoundly affected the degree of neck injury suffered by the driver, whereas the most apparent effect of the change in force-limiting A was on the legs and head. Changing the pre-tightening time C of the seat belt had a clear protective effect on the thorax. The head and chest were the least sensitive to changes in seat belt pre-tensioned force B. In addition, changing force-limiting A and pre-tightening time C caused the least injury to the neck and legs, respectively.
(3) Under the optimized seat belt variables at 4751.618 N, 2,451.839 N, and 17.554 ms for force-limiting A, pre-tensioned force B, and pre-tensioned time C, the head injury HIC and skull von Mises stress decreased by 37.5% and 36.9%, respectively. $N_{ij}$ and cancellous bone stress injuries were reduced by 32.3% and 30.8%, respectively. Moreover, $C_{3ms}$ and $C_{comp}$ were reduced by 37.0% and 37.3%, respectively. Thigh axial force $F_{L}$ was decreased by 8.3%, and weight injury was reduced by 35.1%. The pre-tensioned force-limiting seat belt has great potential for increasing driver protection in small offset collisions.

## Data Availability

The original contributions presented in the study are included in the article/Supplementary Material; further inquiries can be directed to the corresponding authors.

## References

[B1] Albanese B. Bohman K. Bilston L. Koppel S. Brown J. Olivier J. (2020). Influence of child restraint system design features on comfort, belt fit and posture. Saf. Sci. 128, 104707. 10.1016/j.ssci.2020.104707

[B2] Anghileri M. Chirwa E. C. Lanzi L. Mentuccia F. (2005). An inverse approach to identify the constitutive model parameters for crashworthiness modelling of composite structures. Compos. Struct. 68 (1), 65–74. 10.1016/j.compstruct.2004.03.001

[B3] Cao L. B. Ouyang Z. G. Xu Z. Zhang G. J. (2016). Research on the optimization of reversible restraint systems. J. Mech. Eng. 52 (10), 133–141. 10.3901/JME.2016.10.133

[B4] Chan H. S. Liu Y. K. (1974). The asymmetric response of a fluid-filled spherical shell--a mathematical simulation of a glancing blow to the head. J. Biomechanics 7 (1), 43–59. 10.1016/0021-9290(74)90069-4 4820651

[B5] Chi W. C. Geng S. (2019). Research of seat in whiplash based on THUMS human model. Intern. Combust. Engine & Parts 24 (3), 5–7. 10.19475/j.cnki.issn1674-957x.2019.24.003

[B6] Cho Y. B. Bae C. H. Suh M. W. Sin H. C. (2006). A vehicle front frame crash design optimization using hole-type and dent-type crush initiator. Thin-Walled Struct. 44 (4), 415–428. 10.1016/j.tws.2006.03.011

[B7] Deb K. Pratap A. Agarwal S. Meyarivan T. (2002). A fast and elitist multiobjective genetic algorithm: NSGA-II. IEEE Trans. Evol. Comput. 6 (2), 182–197. 10.1109/4235.996017

[B8] Duan A. W. Zhou M. X. Qiu J. L. Feng C. J. Yin Z. Y. Li K. (2020). A 6-year survey of road traffic accidents in southwest China: Emphasis on traumatic brain injury. J. Saf. Res. 73, 161–169. 10.1016/j.jsr.2020.02.010 32563388

[B9] Gao D. W. Zhang N. Chen H. F. (2019). Prediction accuracy of collision indicators for minibuses based on an approximate model. J. Highw. Transp. Res. Dev. 13 (1), 94–103. 10.1061/jhtrcq.0000671

[B10] Ge R. H. Zhang S. X. Hong L. (2017). Optimization of front-row occupant restraint system with NSGA-Ⅱ genetic algorithm. Auto-motive Eng. 39, 1382–1389. 10.19562/j.chinasae.Qcgc.2017.12.005

[B11] Gu K. L. Ge C. Y. Zhang R. (2020). Correlation analysis and structure optimization of seatbelt anchorage strength for Vehicle. Sci. Technol. Eng. 23 (2), 16–18. 10.3969/j.issn.1674-6546.2020.02.005

[B12] Hao Y. Yang S. Han F. F. Ren P. F. He N. Fang R. (2018). Analysis of the influence of the seatbelt fire to the chest damage in mid-low speed frontal impact. Automob. Appl. Technol. 2, 71–74. 10.16638/j.cnki.1671-7988.2018.02.024

[B13] Hassan M. T. Z. Meguid S. A. (2018). Effect of seat belt and head restraint on occupant's response during rear-end collision. Int. J. Mech. Mat. Des. 14 (2), 231–242. 10.1007/s10999-017-9373-6

[B14] Hu Y. Z. Gan S. Liu X. Lian G. J. Zhou L. Liu Z. C. (2018). Deformation control and structure crashworthiness optimization for a certain minivan in the event of a frontal collision. Sci. Technol. Eng. 18 (18), 105–111. 10.3969/j.issn.1671-1815.2018.18.016

[B15] Hu Y. Z. Zeng B. Q. Xie S. G. (2011). The simulation and analysis of automobile safety based on IS-dyna and HyperWorks. Beijing: Tsinghua University press.

[B16] Kallieris D. Rizzetti A. Mattern R. (1997). Some observation to the skull-brain trauma. Advis. Group Aerosp. Res. Dev. Conf. 597, 1–4.

[B17] Kuppa S. Wang J. Haffner M. Eppinger R. (2001). Lower extremity injuries and associated injury criteria. Proc. 17th ESV Conf. 457, 1–15.

[B18] Lei C. Yin Z. Y. (2016). Study of driver injury based on reconstruction of minibus collisions. J. Chongqing Univ. Technol. Nat. Sci. 30 (2), 71–76. 10.3969/j.issn.1674-8425(z).2016.02.013

[B19] Li W. J. Li G. Liu F. J. (2015). Frontal structure safety analysis of minibuses based on Chinese in-depth accident studies. Int. Conf. Tranportation Eng. 0, 2973–2978. 10.1061/9780784479384.379

[B20] Liu S. X. Yang X. J. Cui J. G. Yin Z. Y. (2017a). A novel pixel-based method to estimate the instantaneous velocity of a vehicle from CCTV images. J. Forensic Sci. 62 (4), 1071–1074. 10.1111/1556-4029.13381 28066883

[B21] Liu X. Wu G. Yin L. Y. (2016). Optimal design of a seat belt restraint system based on approximate model management. J. Vib. Shock 35 (6), 132–136. 10.13465/j.cnki.jvs.2016.06.024

[B22] Liu Y. Y. Lu J. Yuan X. C. Du B. T. Yue P. (2017b). Effects of steering column characteristic on driver's injury. Sci. Technol. Eng. 25 (2), 47–50. 10.3969/j.issn.1674-6546.2017.02.011

[B23] Lu F. Zhang J. Y. Ma Y. Liu J. Y. Ye W. T. (2016). The analysis and application of the neck mechanical response of rear seat dummy in frontal crash. Automot. Eng. 38 (7), 828–834. 10.3969/j.issn.1000-680X.2016.07.007

[B24] Magee C. L. Thornton P, H. (1978). Design considerations in energy absorption by structural collapse. Warrendale, Pennsylvania, United States: Society of Automotive Engineers, 55–60. 10.4271/780434

[B25] Marjoux D. Baumgartner D. Deck C. Willinger R. (2008). Head injury prediction capability of the HIC, HIP, SIMon and ULP criteria. Accid. Analysis Prev. 40 (3), 1135–1148. 10.1016/j.aap.2007.12.006 18460382

[B26] Mertz H. J. Prasad P. Irwin A. L. (1997). Injury risk curves for children and adults in frontal and rear collisions. Proc. 41 st Stapp Car Crash Conf. 41, 973318–973330. 10.4271/973318

[B27] Ovesy H. R. Masjedi P. K. (2014). Investigation of the effects of constitutive equations on the free vibration behavior of single-celled thin-walled composite beams. Mech. Adv. Mater. Struct. 21 (10), 836–852. 10.1080/15376494.2012.725266

[B28] Pappachan B. Alexander M. (2012). Biomechanics of cranio-maxillofacial trauma. J. Maxillofac. Oral Surg. 11, 224–230. 10.1007/s12663-011-0289-7 23730074 PMC3386400

[B29] Park G. J. (2011). Technical overview of the equivalent static Loads method for non linear static response structural optimization. Struct. Multidiscipl. Optim. 43 (3), 319–337. 10.1007/s00158-010-0530-x

[B30] Prochowski L. Dębowski A. Żuchowski A. Zielonka K. (2016). Evaluation of the influence of velocity on dynamic passenger loads during a frontal minivan impact against an obstacle. IOP Conference Series: Materials Science and Engineering. IOP Conf. Ser. Mat. Sci. Eng. 148 (1), 012024–24. 10.1088/1757-899X/148/1/012024

[B31] Schaefer K. E. Mcnulty W. P. Carey C. Liebow A. (1958). Mechanisms in development of interstitial emphysema and air embolism on decompression from depth. J. Appl. Physiology 13 (1), 15–29. 10.1152/jappl.1958.13.1.15 13563337

[B32] Shi L. L. Lei C. Li K. Fu S. S. Wu Z. W. Yin Z. Y. (2016). Finite element simulation of lower limb injuries to the driver in minibus frontal collisions. Chin. J. Traumatology 19 (003), 146–150. 10.1016/j.cjtee.2016.01.015 PMC4908227 27321294

[B33] Shigeta K. Kitagawa Y. Yasuki T. (2009). Development of next generation human body FE model capable of organ injury prediction. Proc. 21st Annu. Enhanc. Saf. Veh. 09, 0111. 10.1016/0921-8696(89)90628-2

[B34] Tian G. H. Cheng H. D. Shun P. Y. Wu X. L. (2016). Simulation of dual-stage gas generator airbag. Sci. Technol. Eng. 32 (11), 23–25. 10.3969/j.issn.1674-6546.2016.11.005

[B35] Viano D. C. Arepally S. (1990). Assessing the safety performance of occupant restraint systems. Stapp Car Crash Conf. 27, 1913–1939. 10.4271/902328

[B36] Wang F. Wu J. Z. Hu L. Yu C. Wang B. Y. Huang X. Q. (2022b). Evaluation of the head protection effectiveness of cyclist helmets using full-scale computational biomechanics modelling of cycling accidents. J. Saf. Res. 80, 109–134. 10.1016/j.jsr.2021.11.005 35249593

[B37] Wang F. Yin J. J. Hu L. Wang M. L. Liu X. Miller K. (2022a). Should anthropometric differences between the commonly used pedestrian computational biomechanics models and Chinese population be taken into account when predicting pedestrian head kinematics and injury in vehicle collisions in China? Accid. Analysis Prev. 173, 106718. 10.1016/j.aap.2022.106718 35640364

[B38] Wang W. W. Dai S. J. Zhao W. Z. Wang C. Y. Ma T. Chen Q. Y. (2020). Reliability-based optimization of a novel negative Poisson's ratio door anti-collision beam under side impact. Thin-Walled Struct. 154 (1), 106863. 10.1016/j.tws.2020.106863

[B39] Watanabe R. Miyazaki H. Kitagawa Y. Yasuki T. (2012). Research of collision speed dependency of pedestrian head and chest injuries using human FE model (THUMS version 4). Accid. Reconstr. J. 22, 11–0043. 10.4271/2012-22-0007 23625564

[B40] Wen H. Lin L. Y. Chen D. Q. Wu F. Zhu L. L. (2011). Features of survived casualties and treatment after “July 23” EMU railway accident at Wenzhou station. Chin. J. Emerg. Med. 20 (12), 1248–1250.

[B41] Willinger R. Baumgartner D. Chinn B. Neale M. (2000). Head tolerance limits derived from numerical replication of real world accidents. Proc. Int. Res. Counc. Biomechanics Inj. Conf. 28, 209–221.

[B42] Xiao L. Li L. Duan D. W. Liu Y. B. (2018). Modification design of a sedan based on 25% overlap frontal crash test. Automot. Eng. 040 (002), 184–191. 10.19562/j.chinasae.qcgc.2018.02.011

[B43] Yang W. Xie S. Li H. Chen Z. (2019). Design and injury analysis of the seated occupant protection posture in train collision. Saf. Sci. 117, 263–275. 10.1016/j.ssci.2019.04.028

[B44] Yin H. F. Wen G. L. Liu Z. B. Qing Q. X. (2014). Crashworthiness optimization design for foam-filled multi-cell thin-walled structures. Thin-Walled Struct. 75, 8–17. 10.1016/j.tws.2013.10.022

[B45] Zhai K. N. Li X. Y. Wang Z. Y. Yang S. (2017). Multi-objective optimization of the driver restraint system in the offset crash. Automob. Appl. Technol. 16, 93–95.

[B46] Zhai X. J. Wu Y. Q. Liu W. G. Zhou D. Y. Zhang H. Y. Zhu H. (2015). Optimization of steering column's arrangement angles based on occupant protection. Sci. Technol. Eng. 11 (2), 26–30. 10.3969/j.issn.1674-6546.2015.02.005

